# Reduced *Slc2a4*/GLUT4 expression in subcutaneous adipose tissue of monosodium glutamate obese mice is recovered after atorvastatin treatment

**DOI:** 10.1186/s13098-015-0015-6

**Published:** 2015-03-14

**Authors:** Ana Cláudia Poletto, Aline David-Silva, Aline Pedro de Melo Yamamoto, Ubiratan Fabres Machado, Daniela Tomie Furuya

**Affiliations:** Department of Physiology and Biophysics, Institute of Biomedical Sciences, University of São Paulo, Av. Prof. Lineu Prestes 1524, 05508-900 São Paulo, Brazil

**Keywords:** GLUT4, Statin, Insulin resistance, Obesity, Inflammation, Subcutaneous adipose tissue

## Abstract

**Background:**

Decreased expression of glucose transporter protein GLUT4, encoded by the solute carrier 2A4 (*Slc2a4*) gene, is involved in obesity-induced insulin resistance. Local tissue inflammation, by nuclear factor-κB (NFκB)-mediated pathway, has been related to *Slc2a4* repression; a mechanism that could be modulated by statins. Using a model of obesity with insulin resistance, this study investigated whether (1) inflammatory markers and *Slc2a4* expression are altered; (2) atorvastatin has beneficial effects on inflammation and *Slc2a4* expression; and (3) inhibitor of NFκB (IKK)/NFκB pathway is involved in subcutaneous adipose tissue (SAT).

**Findings:**

Obese mice showed insulin resistance, decreased expression of *Slc2a4* mRNA (66%, *P* < 0.01) and GLUT4 protein (30%, *P* < 0.05), and increased expression of interleukin 6 (*Il6*) mRNA (44%, *P* < 0.05) in SAT. Obese mice treated with atorvastatin had enhanced *in vivo* insulin sensitivity, besides increased *Slc2a4/*GLUT4 expression and reduced *Il6* expression in SAT. No alterations of tumor necrosis factor-α, interleukin 1β and adiponectin expression or IKKα/β activity in SAT of obese mice or obese mice treated with atorvastatin were observed.

**Conclusions:**

Atorvastatin has beneficial effect upon glycemic homeostasis, which may be related to its positive impact on *Il6* and *Slc2a4*/GLUT4 expression in SAT.

## Introduction

The glucose transporter protein GLUT4 is responsible for insulin-mediated glucose uptake in adipose tissue and skeletal muscle, and plays an important role in glycemic homeostasis [1]. In adipocytes, several transcriptional factors regulate *Slc2a4* gene, which encodes for GLUT4 protein, including the nuclear factor-κB (NFκB) [2-4].

Obesity is associated with insulin resistance and reduced *Slc2a4/*GLUT4 expression in both muscle and fat [2,5]. On the other hand, studies on transgenic mice have pointed out that overexpression of GLUT4 in fat enhances *in vivo* glucose tolerance and insulin sensitivity [6]. Furthermore, since the identification of proinflammatory cytokine TNF-α in fat [7] and its relationship to insulin resistance [8], obesity has been closely related to a low grade chronic inflammatory state.

Several studies have demonstrated that statins exert pleiotropic actions besides cholesterol lowering. Recently, some studies have reported evidences for anti-inflammatory and insulin sensitizing effects of statin in visceral adipose tissue (VAT) of glutamate monosodium-induced obese mice [2,9]. In addition, there are evidences that IKK/NFκB pathway is involved in the *Slc2a4* gene expression in VAT [2].

Not only VAT but also subcutaneous adipose tissue (SAT) have been associated to insulin resistance [10]. Considering that SAT is responsible for most of systemic free fatty acids, which are known to induce peripheral insulin resistance, SAT could have more impact on insulin resistance than VAT [11]. Additionally, it is important to understand molecular differences between VAT and SAT, because these fat depots have different biological properties, regarding glucose and fat cell metabolism [12,13]. The present study addresses the effects of atorvastatin on glucose disposal and inflammation focusing on SAT. For that purpose, we used a previous tested model of obesity and insulin resistance, and an anti-inflammatory and insulin sensitizing treatment [2,5].

## Methods

### Reagents

Monosodium glutamate (MSG) was obtained from Sigma (St. Louis, MO) and atorvastatin, from Pfizer (Guarulhos, SP, Brazil). Trizol, DNaseI and Platinum SYBR Green qPCR SuperMix UDG were obtained from Invitrogen (Carlsbad, CA). GoTaq DNA Polymerase was obtained from Promega (Madison, WI). Antibodies for phosphorylated IKK-α/β (Ser180/Ser181) were obtained from Cell Signaling (Beverly, MA) and for GLUT4 from Chemicon (Temecula, CA). Plasma glucose, AST and ALT were assayed with kits purchased from CELM (São Paulo, SP). Glycemia for intravenous insulin tolerance test (IVITT) was measured with a glucometer (Precision QID, Medisense, Bedford, MA).

### Animals treatments

Obesity induction in male offspring mice (CD1) was carried out by subcutaneous injections of MSG (2 mg/g body weight) from days one to five, and on day seven after birth [2]. Control mice were injected with saline solution. Animals were weaned and allowed free access to standard rodent chow and water *ad libitum* until 19 weeks of age, when atorvastatin treatment of obese mice started. Atorvastatin was given in chow (0.1% w/w) for four weeks. At the end of treatment (23-week-old mice) the obesity degree was estimated by Lee obesity index [body weight (g)^1/3^/nasoanal length (cm)], and subcutaneous adipose tissue (SAT) from abdominal, lateral and dorsal regions, as well as blood samples were collected under anesthesia (50 mg/kg b.w. sodium pentobarbital, *i.p.*). Blood samples for glucose, insulin, cholesterol, AST and ALT quantification were collected from retro-ocular bleeding. For ITT experiments, another group of animals was used and blood samples were collected from the tail vein. Mice were euthanized with an overdose of sodium pentobarbital. All procedures were approved by the Ethical Committee for Animal Research of the Institute of Biomedical Sciences, University of São Paulo (123/2005).

### Plasma analysis

Levels of plasma glucose, insulin, cholesterol, activity of aspartate aminotransferase (AST) and alanine aminotransferase (ALT) were measured as previously described [14,15] after 4-hour restricted feeding.

Insulin sensitivity was analyzed by measuring the glucose disappearance constant (kITT) during the intravenous insulin tolerance test (IVITT) [14].

### GLUT4 protein and *Slc2a4* mRNA analysis

GLUT4 was analyzed in SAT by Western blotting and *Slc2a4* mRNA by real-time PCR as previously described [4]. For Western blotting, protein-loaded control was checked by analyzing post-transferring Coomassie-stained gels [16]. For real-time PCR, several housekeeping genes, such as mouse *Rplp0 (36B4)*, *Gapdh* and *Actb* were tested. *Rplp0* was used for normalization. The primer sequences of the following genes, mouse *Slc2a4* [GenBank: NM_009204], *Il6* [NM_031168], *Tnf* [NM_013693], *Il1b* [NM_008361], *Rplp0* [NM_007475], *Gapdh* [NM_008084] were previously described [2,3]. Other primer sequences are as follows: *Adipoq* [NM_009605] (*fw*, 5′-TGGATCTGACGACACCAAAA-3′; *rv*, 5′-ATCCAACCTGCACAAGTTCC-3′) and *Actb* [NM_007393] (*fw*, 5′-ACTGGGACGACATGGAGAAG-3′; *rv*, 5′-GGGGTGTTGAAGGTCTCAAA-3′).

### Phosphorylated and total IKK content

SAT was homogenized in ice-cold extraction buffer [100 mM Tris (pH 7.4), 10 mM EDTA, 10 mM sodium pyrophosphate, 100 mM sodium fluoride, 10 mM sodium vanadate, 2 mM PMSF, 1% Triton X-100, 0.01 mg/mL aprotinin] and centrifuged at 15000 g, 4°C, for 20 min. The supernatants were used to evaluate phosphorylated IKK-α/β and total IKK-β content, which were assayed by Western blotting using anti-phospho-IKK-α (Ser180)/β(Ser181) and anti-IKK-β antibody; respectively (1:1000), followed by standard chemiluminescence detection, and normalization using total protein analysis of post-transferring Coomassie-stained gels [16].

### Statistical analysis

All data are expressed as means ± S.E. Comparison of the means were performed by one-way analysis of variance (ANOVA), with Student–Newman–Keuls as a post hoc test.

## Results

Although untreated obese mice (OB) and atorvastatin-treated obese mice (OBA) showed similar body weight to that observed in control mice (CTL), the significant higher Lee obesity index indicated that both groups were obese (Table 1). Besides, both OB and OBA showed augmentation of absolute (293% and 241% *vs* CTL, *P* < 0.001, respectively) and relative (171% and 132% *vs* CTL, *P* < 0.01, respectively) SAT weight when compared to CTL (Table 1).

**Table 1:** **Characteristics and metabolic profile of the mice under investigation**

	**CTL**	**OB**	**OBA**
Body weight (g)	32.9 ± 0.6	32.5 ± 0.8	32.8 ± 0.5
Lee Obesity Index (x 100)	33.1 ± 0.5	36.8 ± 0.8 **	36.0 ± 0.4**
Absolute SAT weight (g)	0.43 ± 0.08	1.69 ± 0.14 ***	1.47 ± 0.17***
Relative SAT weight (x 100) (g)	1.92 ± 0.62	5.22 ± 0.48**	4.47 ± 0.52**
Absolute VAT weight (g)	0.21 ± 0.01	1.31 ± 0.03 ***	1.35 ± 0.13 ***
Relative VAT weight (x100) (g)	0.64 ± 0.04	4.04 ± 0.16 ***	4.09 ± 0.3 ***
Plasma cholesterol (mmol/L)	1.87 ± 0.09	1.88 ± 0.13	1.33 ± 0.05** ##
Plasma glucose (mmol/L)	8.83 ± 0.27	8.4 ± 0.52	7.8 ± 0.05
Plasma insulin (pmol/L)	532.6 ± 28.5	761.8 ± 19.2***	640.3 ± 10.1* ##
kITT (%/min)	4.84 ± 0.32	2.96 ± 0.30*	4.67 ± 0.62#
Plasma AST activity (U/L)	19.6 ± 2.1	20.6 ± 2.0	21.5 ± 0.5
Plasma ALT activity (U/L)	12.6 ± 0.7	14.6 ± 1.6	15.9 ± 0.4

SAT, subcutanous adipose tissue, VAT, visceral adipose tissue, kITT, glucose disappearance constant obtained in the intravenous insulin tolerance test. Data from plasma cholesterol, glucose and insulin concentration and kITT were obtained from mice subjected to 4-hour food deprivation. Data are means S.E. of 4 to 6 (morphological parameters), and 3 to 7 (metabolic hormonal parameters) animals. *P < 0.05, **P < 0.01 and ***P < 0.001 *vs*. CTL; #*P* < 0.05 and ##*P* < 0.01 *vs*. OB, One-way analysis of variance and Student–Newman–Keuls post hoc test.

**Figure 1:**
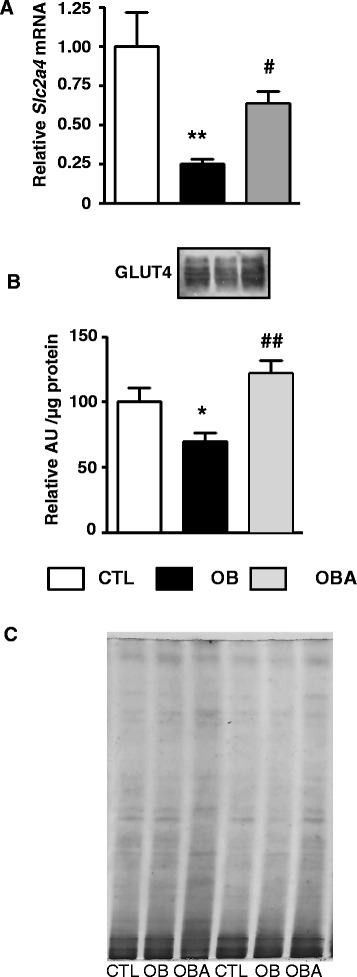
***Slc2a4*** **mRNA (A) and GLUT4 protein (B) expression in subcutaneous white adipose tissue.** Mice were separated into several groups: lean untreated (CTL, white bars), obese untreated (OB, black bars) or obese atorvastatin-treated (OBA, gray bars) mice. In **(A)** and **(B)**, relative values of mRNA or protein content (bottom) are shown. In **B** (top), image of a typical experiment. *Slc2a4* mRNA expression was analyzed by Real-time PCR, normalized by *Rplp0* **(A)**, and GLUT4 protein expression by Western blotting **(B)**, normalized by total protein analysis with Coomassie Blue-stained gel **(C)**. Data are expressed as means ± S.E. of 5–6 (*Slc2a4* mRNA) and 5–6 (GLUT4 protein) animals per group. *P < 0.05 and **P < 0.01 *vs*. CTL; # *P* < 0.05 and ## *P* < 0.01 *vs*. OB; ANOVA and Student–Newman–Keuls post hoc test.

OB presented hyperinsulinemia and decreased glucose disappearance constant (38% *vs* CTL, *P* <0.05) in IVITT, depicting the whole body insulin-resistant condition (Table 1). Moreover, as evident in Figure 1, the expression of *Slc2a4* mRNA and GLUT4 protein in SAT of OB was reduced (66% and 30% *vs* CTL, *P* < 0.01 and *P* < 0.05, respectively), indicating the participation of this territory in the whole body glycemic homeostasis.

Interestingly, atorvastatin treatment did not only reduce plasma cholesterol (Table 1), but also had a positive impact on glucose metabolism, reducing the level of insulinemia and increasing the insulin sensitivity of OBA as measured by IVTT (Table 1). Atorvastatin also restored the expression of *Slc2a4* mRNA and GLUT4 protein in SAT of obese mice (Figure 1). Additionally, the drug did not alter the plasma activity of AST and ALT, indicating no apparent hepatotoxicity (Table 1).

**Figure 2:**
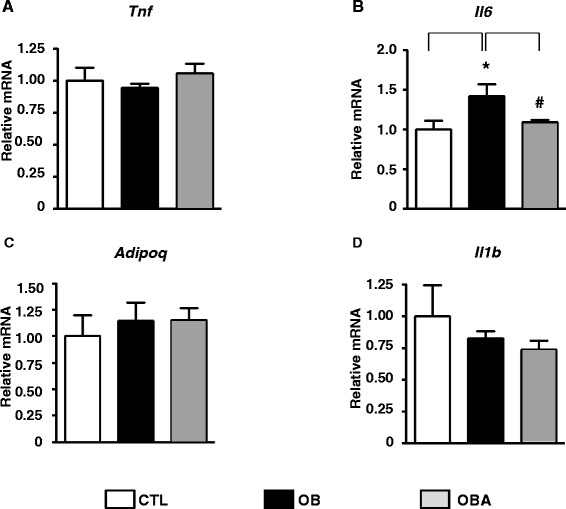
***Tnf*** **(A),** ***Il6*** **(B),** ***Adipoq*** **(C) and** ***Il1b*** **(D) mRNA content in subcutaneous adipose tissue.** Mice were separated into several groups: lean untreated (CTL, white bars), obese untreated (OB, black bars) or obese atorvastatin-treated (OBA, gray bars) mice. The mRNA was analyzed by real-time PCR, and *Rplp0* gene was used for normalization. The values are means ± S.E. of 6 animals per group. *P < 0.05 *vs*. CTL; #*P* < 0.05 *vs*. OB; ANOVA and Student-Newman-Keuls post hoc test.

In order to verify the effect of obesity and atorvastatin treatment upon inflammation in SAT, the expression of some cytokines were investigated. OB showed increased *Il6* mRNA expression in SAT (44% *vs* CTL, *P* < 0.05), but unchanged *Tnf*, *Il1b* or *Adipoq* gene expression (Figure 2). Moreover, atorvastatin treatment was able to drastically reduce *Il6* mRNA expression (44% *vs* OB, *P* < 0.05) in SAT (Figure 2).

**Figure 3:**
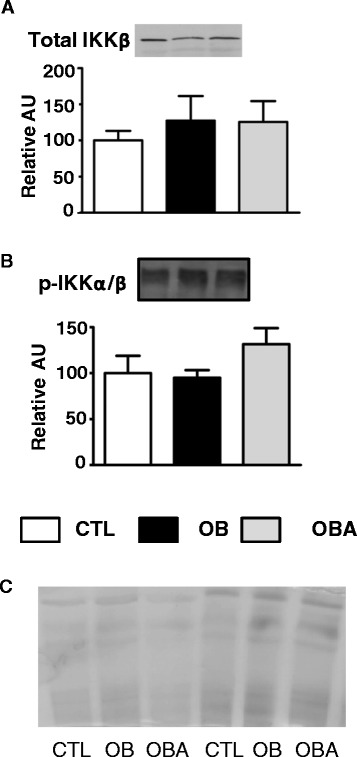
**Total IKK-β and phosphorylated IKKα/β in subcutaneous adipose tissue.** Mice were separated into several groups: lean untreated (CTL, white bars), obese untreated (OB, black bars) or obese atorvastatin-treated (OBA, gray bars) mice. In **(A)** and **(B)**, on top, images of one typical experiment; and, on bottom, relative values of total IKK-β and phosphorylated IKKα/β protein content are shown. Normalization was performed by total protein analysis with Coomassie Blue-stained gel **(C)**. Data are means ± S.E. of 4–7 animals per group.

The IKK/NFκB pathway was accessed in SAT. Total IKK-β content and phosphorylated IKK-α/β in SAT of OB and OBA were unaltered when compared to CTL (Figure 3).

## Discussion

The current study demonstrated that atorvastatin treatment restores GLUT4 protein and mRNA expression in SAT of OB, contributing to the amelioration of whole-body insulin resistance.

It has been reported that atorvastatin has beneficial impacts on inflammation and glucose metabolism. Our laboratory recently demonstrated that OBA had reduced circulating cytokines and reduced cytokines expression in VAT, which resulted in insulin sensitizing effects [2]. SAT and VAT show distinct physiological characteristics such as cytokine expression [17] and insulin signaling [18]. Therefore, by using an established model of obesity and insulin resistance, and an anti-inflammatory treatment (atorvastatin), this study analyzed the SAT of obese mice.

Many reports have shown that reduced GLUT4 expression is related to insulin resistance, and improvement of GLUT4 content is related to increased insulin sensitivity [2,5,19]. Moreover, mice with adipose-selective reduction of GLUT4 have a striking reduction in glucose uptake by adipocytes [20]. In contrast, overexpression of GLUT4 in adipose tissue increases glucose influx into adipocytes [6]. The present data clearly show that obese mice developed insulin resistance which was accompanied by reduced *Slc2a4* mRNA and protein content in SAT. It has been reported that *Slc2a4* mRNA [21] and protein [22] expression is reduced in SAT of obese women. Regarding SAT of obese mice, this is the first report investigating *Slc2a4* expression. It has been reported that OB mice present reduced *Slc2a4* mRNA and GLUT4 protein expression in VAT [2,5]. Therefore, it can be assumed that the reduction of *Slc2a4*/GLUT4 expression in both SAT and VAT contributes to the whole-body insulin resistance in this animal model of obesity. On the other hand, atorvastatin was able to restore *Slc2a4* mRNA and protein content not only in VAT [2], but also in SAT of OB, contributing for amelioration of insulin resistance.

Obesity is considered as a low grade chronic inflammatory state [2,23]. OB showed increased *Il6* expression in SAT, but showed no alteration in *Tnf*, *Il1b* and *Adipoq* expression. The literature concerning the impact of obesity on *Tnf* expression in mice SAT is contentious [24,25]. As to *Il1b* expression in SAT, a single study shows reduction in obese mice [25]. Furthermore, few reports have shown *Il6* increase in VAT of obese mice [2,17], but to date there has been no report about obesity impact on *Il6* expression in SAT of mice. Taking into account that IL-6 can reduce *Slc2a4*/GLUT4 expression in adipocytes [26], the present findings point out that IL-6 may be an important repressor of the *Slc2a4* gene in SAT but the involved mechanisms still needs clarification.

Finally, we are, for the first time, reporting that IKK content and phosphorylation are not altered in SAT of OB. IKK/NFκB negatively modulates the expression of *Slc2a4* gene [2-4,27], and we have demonstrated that the reduced expression of *Slc2a4*/GLUT4 in VAT of OB correlates to enhanced IKK phosphorylation [2]. However, in SAT, the present findings could not confirm the involvement of this inflammatory pathway in the modulation of *Slc2a4* gene.

In conclusion, *Slc2a4* expression in SAT is reduced in OB, which contributes to the impairment of glycemic homeostasis. Atorvastatin treatment improves insulin resistance which may be related to its positive impact on *Il6* and *Slc2a4*/GLUT4 expression in SAT.

## Abbreviations

*Actb*: Beta-actin gene
*Adipoq*: Adiponectin gene
ALT: Alanine aminotransferase
AST: Aspartate aminotransferase
CTL: Control mice
*Gapdh*: Glyceraldehyde-3-phosphate dehydrogenase gene
GLUT4: Glucose transporter 4
IKK: Inhibitor of nuclear factor kappa-B kinase
IL-1β: Interleukin-1 beta
*Il1b*: Interleukin 1 beta gene
IL-6: Interleukin-6
*Il6*: Interleukin 6 gene
IVITT: Intravenous insulin tolerance test
kITT: Glucose disappearance constant
NFκB: Nuclear factor kappa-B
OB: Untreated obese mice
OBA: Atorvastatin-treated obese mice
*Rplp0*: Ribosomal protein, large, P0 gene
SAT: Subcutaneous adipose tissue
*Slc2a4*: Solute carrier family 2 (facilitated glucose transporter), member 4
TNF-α: Tumor necrosis factor alpha
*Tnf*: Tumor necrosis factor alpha gene
VAT: visceral adipose tissue

## References

[1] Kahn BB (1996). Lilly lecture 1995. Glucose transport: pivotal step in insulin action. Diabetes.

[2] Furuya DT, Poletto AC, Favaro RR, Martins JO, Zorn TM, Machado UF (2010). Anti-inflammatory effect of atorvastatin ameliorates insulin resistance in monosodium glutamate-treated obese mice. Metabolism.

[3] Furuya DT, Poletto AC, Freitas HS, Machado UF (2012). Inhibition of cannabinoid CB1 receptor upregulates Slc2a4 expression via nuclear factor-κB and sterol regulatory element-binding protein-1 in adipocytes. J Mol Endocrinol.

[4] Furuya DT, Neri EA, Poletto AC, Anhê GF, Freitas HS, Campello RS (2013). Identification of nuclear factor-κB sites in the *Slc2a4* gene promoter. Mol Cell Endocrinol.

[5] Papa PC, Seraphim PM, Machado UF (1997). Loss of weight restores GLUT 4 content in insulin-sensitive tissues of monosodium glutamate-treated obese mice. Int J Obes Relat Metab Disord.

[6] Shepherd PR, Gnudi L, Tozzo EPR, Yang H, Leach F, Kahn B (1993). Adipose cell hyperplasia and enhanced glucose disposal in transgenic mice overexpressing GLUT4 selectively in adipose tissue. J Biol Chem.

[7] Hotamisligil GS, Shargill NS, Spiegelman BM (1993). Adipose expression of tumor necrosis factor-α: direct role in obesity-linked insulin resistance. Science.

[8] Feinstein R, Papa Kanety H, Lunenfeld B, Karasik A (1993). Tumor necrosis factor-α suppresses insulin-induced tyrosine phosphorylation of insulin receptor and its substrates. J Biol Chem.

[9] Zhang N, Huan Y, Huang H, Song G, Sun S, Shen Z (2010). Atorvastatin improves insulin sensitivity in mice with obesity induced by monosodium glutamate. Acta Pharmacol Sin.

[10] Tulloch-Reid MK, Hanson RL, Sebring NG, Reynolds JC, Premkumar A, Genovese DJ (2004). Both subcutaneous and visceral adipose tissue correlate highly with insulin resistance in african americans. Obes Res.

[11] Garg A (2004). Regional adiposity and insulin resistance. J Clin Endocrinol Metab.

[12] Jensen MD (1997). Lipolysis: contribution from regional fat. Annu Rev Nutr.

[13] Giorgino F, Laviola L, Eriksson JW (2005). Regional differences of insulin action in adipose tissue: insights from in vivo and in vitro studies. Acta Physiol Scand.

[14] Furuya DT, Binsack R, Machado UF (2003). Low ethanol consumption increases insulin sensitivity in Wistar rats. Braz J Med Biol Res.

[15] Tomie Furuya D, Binsack R, Onishi ME, Monteiro Seraphim P, Fabres Machado UF (2005). Low ethanol consumption induces enhancement of insulin sensitivity in liver of normal rats. Life Sci.

[16] Ferguson RE, Carroll HP, Harris A, Maher ER, Selby PJ, Banks RE (2005). Housekeeping proteins: A preliminary study illustrating some limitations as useful references in protein expression studies. Proteomics.

[17] Boucher J, Castan-Laurell I, Daviaud D, Guigne C, Buleon M, Carpene C (2005). Adipokine expression profile in adipocytes of different mouse models of obesity. Horm Metab Res.

[18] Laviola L, Perrini S, Cignarelli AC, Leonardini A, De Stefano F, Cuscito M (2006). Insulin signaling in human visceral and subcutaneous adipose tissue in vivo. Diabetes.

[19] Berger J, Biswas C, Vicario PP, Strout HV, Saperstein R, Pilch PF (1989). Decreased expression of the insulin-responsive glucose transporter in diabetes and fasting. Nature.

[20] Abel ED, Peroni O, Kim JK, Kim YB, Boss O, Hadro E (2001). Adipose-selective targeting of the GLUT4 gene impairs insulin action in muscle and liver. Nature.

[21] Veilleux A, Blouin K, Rhéaume C, Daria M, Marette A, Tchemof A (2009). Glucose transporter 4 and insulin receptor substrate-1 messenger RNA expression in omental and subcutaneous adipose tissue in women. Metabolism.

[22] Björnholm M, Al-Khalili L, Dicker A, Näslund E, Rössner S, Zierath JR (2002). Insulin signal transduction and glucose transport in human adipocytes: effects of obesity and low calorie diet. Diabetologia.

[23] Shoelson SE, Lee J, Goldfine AB (2006). Inflammation and insulin resistance. J Clin Invest.

[24] Takeshita S, Kawamura I, Yasuno T, Kimura C, Yamamoto T, Seki J (2001). Amelioration of insulin resistance in diabetic ob/ob mice by a new type of orally active insulin-mimetic vanadyl complex: bis(1-oxy-2-pyridinethiolato)oxovanadium(IV) with VO(S(2)O(2)) coordination mode. J Inorg Biochem.

[25] Juge-Aubry CE, Somm E, Giusti V, Pernin A, Chicheportiche R, Verdumo C (2003). Adipose tissue is a major source of interleukin-1 receptor antagonist: upregulation in obesity and inflammation. Diabetes.

[26] Lagathu C, Bastard JP, Auclair M, Maachi M, Capeau J, Caron M (2003). Chronic interleukin-6 (IL-6) treatment increased IL-6 secretion and induced insulin resistance in adipocyte: prevention by rosiglitazone. Biochem Biophys Res Commun.

[27] Silva JL, Giannocco G, Furuya DT, Lima GA, Moraes PAC, Nachef S (2005). NF-kappaB, MEF2A, MEF2D and HIF1-a involvement on insulin- and contraction-induced regulation of GLUT4 gene expression in soleus muscle. Mol Cell Endocrinol.

